# Inhibition of Polo-like Kinase 1 by HMN-214 Blocks Cell Cycle Progression and Inhibits Neuroblastoma Growth

**DOI:** 10.3390/ph15050523

**Published:** 2022-04-24

**Authors:** Rameswari Chilamakuri, Danielle Crystal Rouse, Saurabh Agarwal

**Affiliations:** Department of Pharmaceutical Sciences, College of Pharmacy and Health Sciences, St. John’s University, New York, NY 11439, USA; rameswari.chilamakuri19@my.stjohns.edu (R.C.); danielle.rouse20@my.stjohns.edu (D.C.R.)

**Keywords:** pediatric cancer, neuroblastoma, HMN-214, cell cycle, PLK1

## Abstract

Polo-like kinase 1 (PLK1) is an essential cell cycle mitotic kinase component that plays an important role in cell cycle progression and has been reported to be involved in various cancers, including neuroblastoma (NB). PLK1 also regulates G2/M transition, chromosomal segregation, spindle assembly maturation, and mitotic exit. NB is an early embryonic-stage heterogeneous solid tumor and accounts for 15% of all pediatric cancer-related deaths. Therefore, we aimed to develop a targeting strategy for PLK1 by repurposing HMN-214 in NB. HMN-214 is a prodrug of HMN-176 and is known to selectively interfere with PLK1 function. In the present study, we performed the transcriptomic analysis of a large cohort of primary NB patient samples and revealed that *PLK1* expression is inversely correlated with the overall survival of NB patients. Additionally, we found that *PLK1* strongly correlates with NB disease and stage progression. HMN-214 significantly inhibited NB proliferation and colony formation in both MYCN-amplified and -nonamplified cell lines in a dose-dependent manner. Furthermore, HMN-214 induces apoptosis and significantly obstructs the cell cycle at the G2/M phase in NB cells by inhibiting multiple cell-cycle-related genes, such as *PLK1*, *WEE1*, *CDK1*, *CDK2*, *Cyclin B1*, *CHK1*, and *CHK2*. HMN-214 significantly inhibits cell cycle regulator CDK1 and the phosphorylation and activation of PLK1 in NB. In the NB 3D spheroid tumor model, HMN-214 significantly and in a dose-dependent manner inhibits spheroid tumor mass and growth. Overall, our study highlights that targeting PLK1 using HMN-214 is a novel therapeutic approach for NB.

## 1. Introduction

Neuroblastoma (NB) is the most prevalent heterogeneous solid tumor that develops during the early embryonic stage from the sympathetic nervous system [1]. NB accounts for 15% of pediatric cancer-related deaths and remains a challenge for pediatric oncologists [2,3]. Despite intensive multimodal therapeutic approaches, such as chemotherapy, radiation, and surgery, the prognosis of high-risk NB patients is abysmal with higher metastatic and relapse conditions [3]. Hence, developing novel therapeutic approaches to improve survival and reduce long-term toxicities are needed to efficiently treat NB.

Tumorigenesis mainly depends on the activation of oncogenes and the inactivation of tumor suppressor genes [4]. Most of these proto-oncogenes and tumor suppressor genes play an important role in regulating the cell cycle progression, and dysregulation of these genes contributes to cancer progression [4]. PLK1 (Polo-like kinase 1) is a member of the polo-like kinase family of serine/threonine protein kinases and plays an important role in regulating the cell cycle. PLK1 contains an N-terminal catalytic domain, C-terminal polo-box domain (PBD), and a phosphopeptide connecting region in the middle that connects both N- and C-terminal domains of the PLK1 [5]. The C-terminal PBD consists of two polo-box structures, an important feature of PLK family proteins, whereas the N-terminal catalytic domain of PLK1 is a Serine/Threonine kinase domain [6]. Upon ligand binding, the PBD dissociates from the catalytic kinase domain and phosphorylates different cellular proteins or different sites of the same protein [7]. Aurora-A kinase phosphorylates and activates the Thr210 loop of PLK1 in the mitotic phase [8]. PLK1 has a strong relationship with numerous cell cycle events such as G2/M transition, chromosomal segregation, spindle assembly maturation, and mitotic exit [9]. PLK1 has a functional niche beyond mitosis, and it regulates DNA damage response, DNA replication, transcription, translation, chromosomes dynamics, and checkpoint adoption [10]. High levels of PLK1 are restricted to actively dividing cells such as embryonic cells and hair follicles [11]. Elevated levels of PLK1 are also observed in different cancers, including NB, breast, colorectal, melanoma, prostate, ovarian, esophageal, and non-small-cell lung cancer [12,13,14,15,16].

In the present study, we evaluated the effects of a small-molecule PLK1 inhibitor HMN-214 on NB growth [17,18]. HMN-214 [{E)-4-{2-[2-(N-acetyl-N-[4-methoxybenzenesulfonyl] amino) stilbazole]}1-oxide] is a prodrug of HMN-176 ((E)-4-{[2-N-[4-methoxybenzenesulfonyl] amino]-stilbazole}1-oxide) and is developed by Nippon Shinyaku Co. Ltd. (Kyoto, Japan). HMN-214 is currently in the early clinical development phase and has shown potent anti-tumor activity against lung, pancreatic, gastric, prostate, and breast cancer models [19,20,21,22]. HMN-214 indirectly inhibits PLK1 by altering spatial distribution, resulting in cell cycle arrest at the G2/M phase, with the destruction of the spindle polar bodies followed by DNA fragmentation [20,22,23,24]. Overall, our results confirmed that HMN-214 inhibits NB proliferation in both 2D cell cultures and 3D spheroid tumor models in a dose-dependent manner by inhibiting the cell cycle progression and inducing apoptosis by inhibiting the phosphorylation of PLK1.

## 2. Results

### 2.1. PLK1 Expression Strongly Correlates with Poor NB Survival

**Figure 1:**
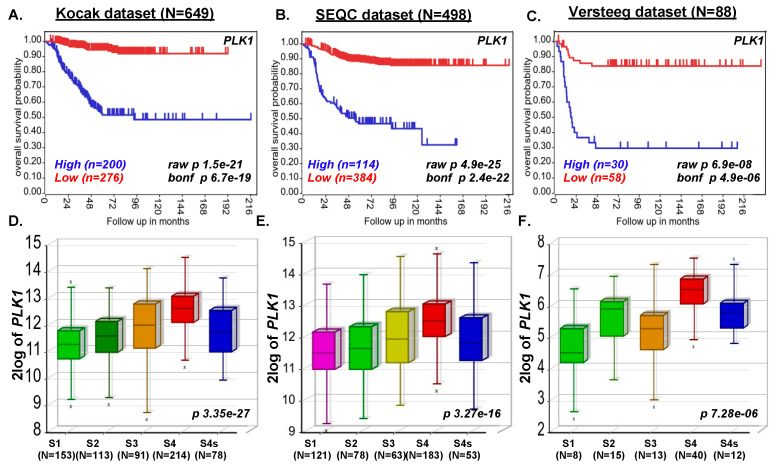
*PLK1* expression correlates with poor overall survival of NB patients. Kaplan–Meier analysis of *PLK1* gene showing poor overall probability of NB patients’ survival. (**A**) Kocak dataset (N = 649 patients). (**B**) SEQC dataset (N = 498 patients). (**C**) Versteeg dataset (N = 88 patients). (**D**–**F**) Correlation of *PLK1* expression with NB stage progression. (**D**) Kocak dataset (**E**) SEQC dataset (**F**) Versteeg dataset. N represents the number of patient samples.

To determine the role of *PLK1* gene expression in NB progression, we investigated the transcriptional data of 1235 NB primary patient samples, including the Versteeg dataset (*n* = 88), Kocak dataset (*n* = 649), and SEQC dataset (*n* = 498). Kaplan–Meier survival analysis revealed that *PLK1* expression inversely correlates with the poor overall survival of NB patients. Higher expression of the *PLK1* gene leads to poor overall survival of NB patients (Kocak *n* = 649, *p* = 1.5 × 10^−21^; SEQC *n* = 498, *p* = 4.9e-25, Versteeg *n* = 88, *p* = 6. × 10^−8^; Figure 1A–C). Furthermore, we observed a significant correlation in the expression of *PLK1* and NB stage progression (Kocak *n* = 649, *p* = 3.35 × 10^−27^; SEQC *n* = 498, *p* = 3.27 × 10^−16^, Versteeg (*n* = 88, *p* = 7.28 × 10^−6^; Figure 1D–F), suggesting that *PLK1* plays a significant role in NB stage progression. We also observed high *PLK1* expression in highly aggressive MYCN-amplified tumors, which correlates with NB disease progression and recurrence in the Versteeg dataset (data not shown). These findings from primary NB patient samples suggest that *PLK1* is a critical prognostic factor for NB disease and stage progression.

### 2.2. HMN-214 Inhibits NB Cell Proliferation and Colony Formation

**Figure 2:**
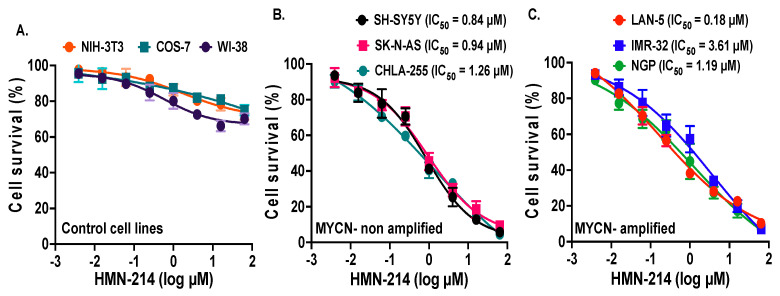
HMN-214 inhibits NB proliferation. Cell proliferation assays in response to HMN-214 using six human NB cell lines and three control fibroblast cell lines. (**A**) Control fibroblast cell lines (WI-38, NIH-3T3, COS-7). (**B**) MYCN-non-amplified cell lines (SH-SY5Y, SK-N-AS, CHLA-255). (**C**) MYCN amplified cell lines (NGP, LAN-5, CHLA-255-MYCN). Respective IC_50_ values are indicated.

**Figure 3:**
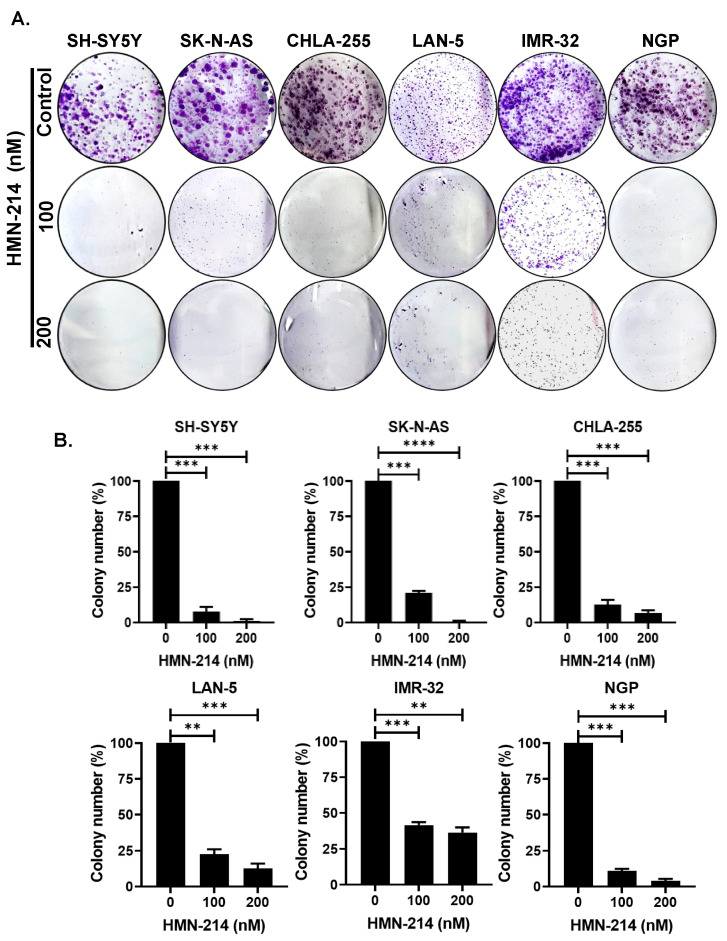
HMN-214 inhibits NB colony formation. Colony formation assay in response to HMN-214 in six NB cell lines. (**A**) Representative images of colony formation assays. (**B**) Quantitative representation of relative inhibition of colony formation. ** *p* < 0.01, *** *p* < 0.001, and **** *p* < 0.0001.

Furthermore, we used a specific small-molecule PLK1 inhibitor, HMN-214, and performed proliferation assays using differentrent NB cell lines. Cytotoxic assays using three MYCN-amplified (NGP, LAN-5, and IMR-32), three MYCN-non-amplified NB cell lines (SH-SY5Y, CHLA-255, and SK-N-AS), and three control fibroblast cell lines (WI-38, NIH-3T3, COS-7) showed that HMN-214 significantly and dose-dependently inhibits NB cell proliferation (Figure 2). Both MYCN-amplified and -non-amplified cell lines showed efficient inhibition in cell proliferation in contrast to control fibroblast cell lines (Figure 2). We further performed the clonogenic assays to validate the anti-proliferative effect of HMN-214. Results showed that HMN-214 significantly inhibits NB colony formation capacity in a dose-dependent manner in treatment groups compared to controls in all six NB cell lines tested (Figure 3). These data indicate that HMN-214 is an effective drug and inhibits NB proliferation, colony formation, and induces cytotoxicity.

### 2.3. HMN-214 Induces Apoptosis and Blocks Cell Cycle Progression in NB

**Figure 4:**
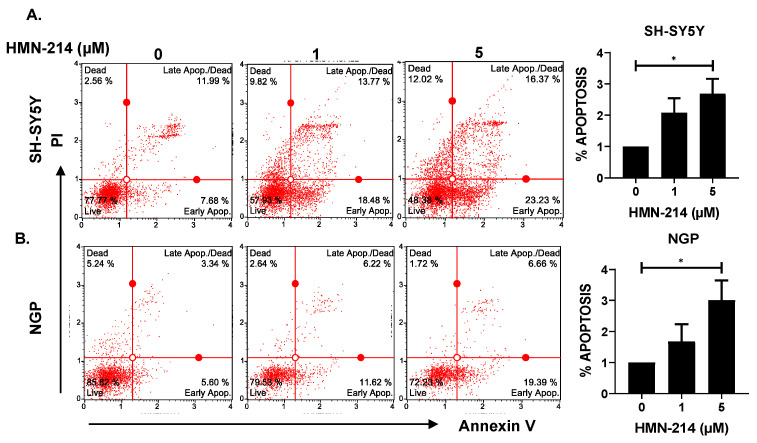
HMN-214 induces apoptosis in NB. Apoptosis assays performed in response to HMN-214 treatment in both MYCN-non-amplified (SH-SY5Y) and MYCN-amplified (NGP) cell line. (**A**,**B**) Representative flow cytometer images and quantitative representation of percentage of early apoptosis. (**A**) SH-SY5Y, (**B**) NGP. * *p* < 0.05.

**Figure 5:**
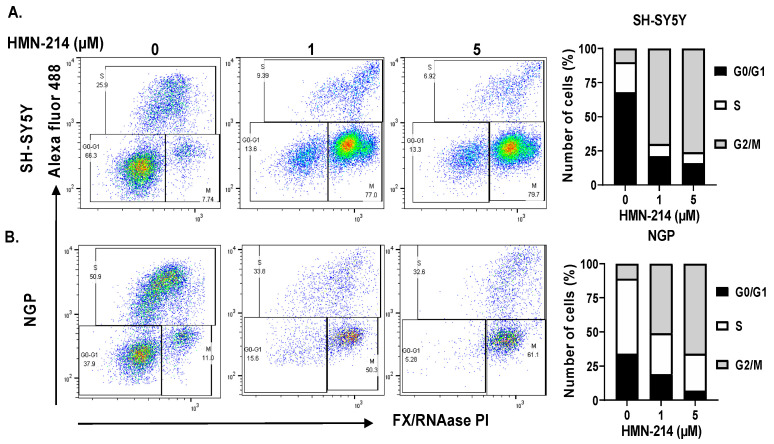
HMN-214 blocks cell cycle in NB. Cell cycle assay performed in response to HMN-214 treatment in both MYCN-non-amplified (SH-SY5Y) and MYCN-amplified (NGP) cell line. (**A**,**B**) Representative flow cytometer images and quantitation of the percentage of cells in different cell cycle phases. (**A**) SH-SY5Y, (**B**) NGP.

HMN-214 is known to inhibit the cell cycle progression of various cancer cells, including breast, colon, and esophageal cancer [21]. We performed apoptosis and cell cycle assays using HMN-214 in both MYCN-amplified (NGP) and MYCN-non-amplified (SH-SY5Y) NB cell lines. Results showed that HMN-214 significantly induces apoptosis in both MYCN-amplified and -non-amplified cell lines in a dose-dependent manner. The percentage of early apoptosis cells was about 3.0 and 3.5 folds higher in response to 5 µM HMN-214 treatment in SH-SY5Y and NGP, respectively, compared to control groups (Figure 4). Furthermore, our cell cycle analysis results revealed that HMN-214 significantly blocks the NB cell cycle progression in a dose-dependent manner by inhibiting the G2/M phase transition (Figure 5). The percentage of cells in the G2/M phase significantly increased by about 10.5 and 6.0 folds higher in response to 5 µM HMN-214 treatment in SH-SY5Y and NGP, respectively, in comparison to control treatment. Additionally, the percentage of cells in the S phase decreased by about 3.7 and 1.5 folds in SH-SY5Y and NGP, respectively, in contrast to controls (Figure 5). Our results confirmed the effect and potency of HMN-214 in inducing apoptosis and arresting the cell cycle progression in NB cells.

### 2.4. HMN-214 Inhibits NB Spheroid Tumor Growth

**Figure 6:**
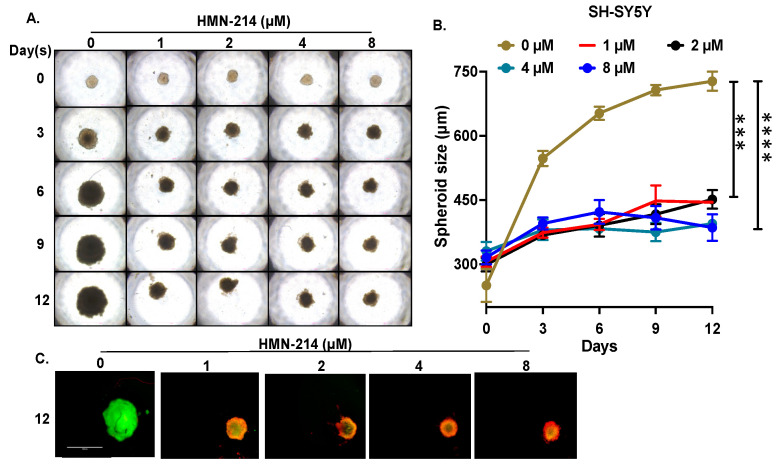
HMN-214 inhibits MYCN-non-amplified SH-SY5Y NB spheroid tumor growth. (**A**) Representative images of SH-SY5Y NB spheroidal tumors. (**B**) Spheroid tumor growth measurements in response to different concentrations of HMN-214 treatment at different days. (**C**) Representative fluorescent images of Day 12 spheroidal images stained with Calcein AM (Green; live cells) and EthD-III (Red; dead cells) fluorescence dyes. *** *p* < 0.001, **** *p* < 0.0001.

**Figure 7:**
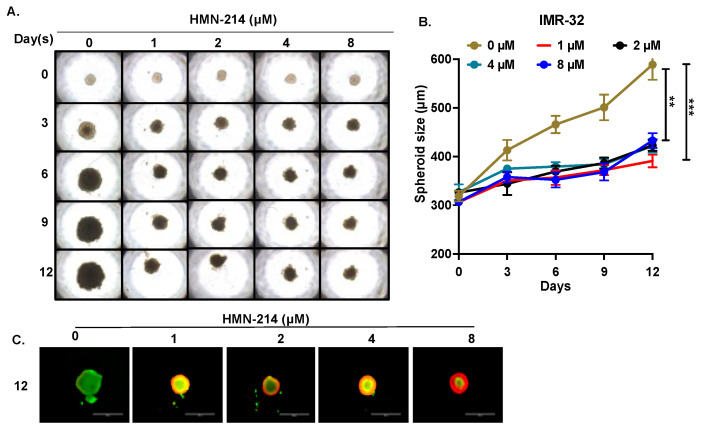
HMN-214 inhibits MYCN-amplified IMR-32 NB spheroid tumor growth. (**A**) Representative images of IMR-32 NB spheroidal tumors. (**B**) Spheroid tumor growth measurements in response to different concentrations of HMN-214 treatment at different days. (**C**) Representative fluorescent images of Day 12 spheroidal images stained with Calcein AM (Green; live cells) and EthD-III (Red; dead cells) fluorescence dyes. ** *p* < 0.01, *** *p* < 0.001.

We further evaluated the anti-proliferative effect of HMN-214 in NB 3D spheroid tumor models that mimic the physiological growth patterns of in vivo solid tumor growth. We developed 3D spheroid tumors using both MYCN-non-amplified (SH-SY5Y) (Figure 6), and MYCN-amplified (IMR-32) cell lines (Figure 7). Our results demonstrate that HMN-214 significantly inhibits NB spheroid tumor growth in a dose-dependent manner, in contrast to controls in both MYCN-amplified and MYCN-non-amplified spheroid tumors (Figure 6A and Figure 7A). We found a two-fold reduction in tumor size with 1 µM of HMN-214 treatment in both SH-SY5Y and IMR-32 spheroid tumors (Figure 6B and Figure 7B). Furthermore, fluorescent staining of terminal day spheroids showed a dose-dependent reduction in the number of live cells and an increase in the number of dead cells, confirming a dose-dependent effect of HMN-214 in inhibiting NB cell growth (Figure 6C and Figure 7C). Our 3D spheroid tumor results demonstrated that HMN-214 significantly induces tumor cell death to inhibit NB spheroid tumor growth in different NB spheroid tumor models.

### 2.5. HMN-214 Inhibits PLK1 Pathway and Cell Cycle Signaling Cascade

**Figure 8:**
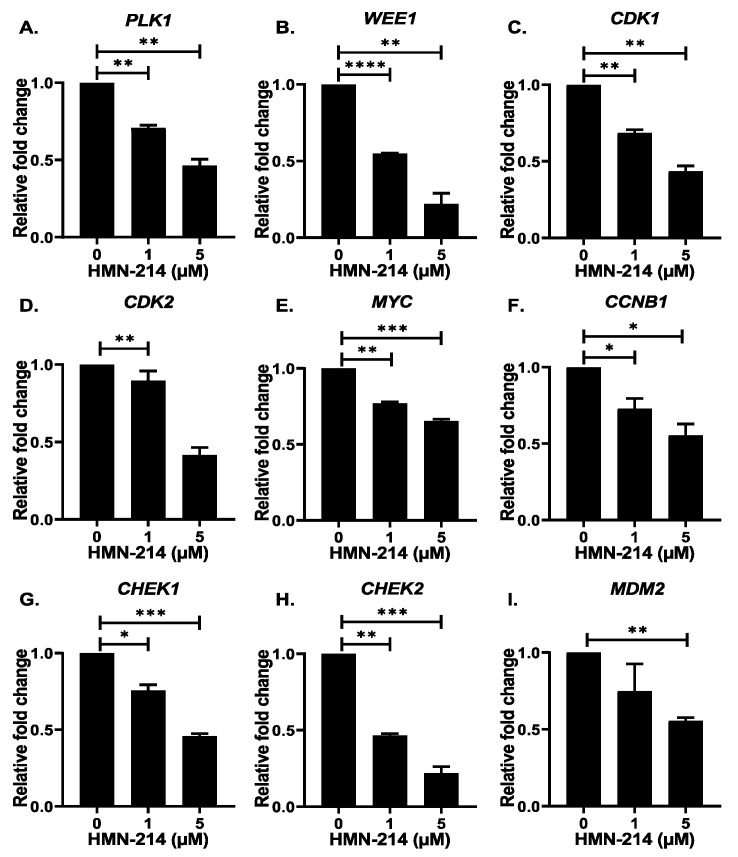
HMN-214 inhibits critical NB regulatory genes. Gene expression analysis of PLK1 pathway and cell cycle regulatory genes in response to HMN-214 in SH-SY5Y cells. (**A**) *PLK1*, (**B**) *WEE1*, (**C**) *CDK1*, (**D**) *CDK2*, (**E**) *MYC*, (**F**) *CCNB1* (Cyclin-B1), (**G**) *CHEK1* (CHK1), (**H**) *CHEK2* (CHK2), and (**I**) *MDM2*. * *p* < 0.05, ** *p* < 0.01, *** *p* < 0.001, **** *p* < 0.0001.

**Figure 9:**
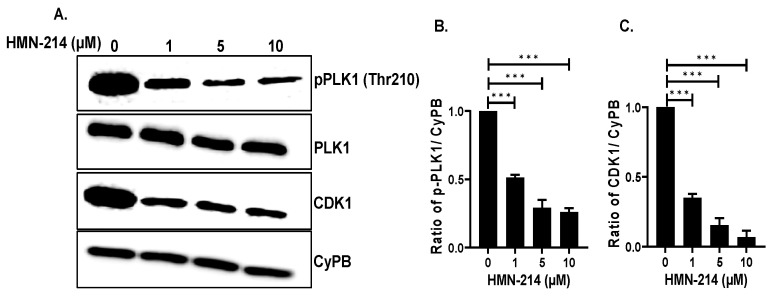
HMN-214 inhibits PLK1 activation. (**A**) Western blot analysis of PLK1, pPLK1 (Thr210), and CDK1 in response to HMN-214 treatment in SH-SY5Y cells. CyPB is used as loading control. (**B**,**C**) Densiometric analysis of (**B**) pPLK1 and (**C**) CDK1. *** *p* < 0.001.

To further evaluate the mechanism of action of HMN-214 in NB cells, we performed gene expression analysis of different NB cell cycle-related genes and Western blot analysis of PLK1 and CDK1. Our RT-qPCR analysis demonstrates that HMN-214 significantly inhibits the mRNA expression of *PLK1* pathway genes, such as *PLK1*, *CCNB1* (Cyclin-B1)*,* and *CDK1*; proto-oncogenes, such as *MYC* and *MDM2*; and cell cycle regulator genes, such as *WEE1*, *CDK2*, *CHEK1* (CHK1), and *CHEK2* (CHK2) in a dose-dependent manner in contrast to controls (Figure 8). Furthermore, our western blot analysis confirmed that HMN-214 significantly and in a dose-dependent manner inhibits phosphorylation of PLK1 at its Thr210 catalytic site (Figure 9A,B), whereas the total PLK1 level remains unaffected. This data further confirms that HMN-214 directly targets the phosphorylation and activation of PLK1. Furthermore, we observed a significant reduction in CDK1 levels in response to HMN-214 treatments (Figure 9A,C). Overall, our molecular assays demonstrated that HMN-214 inhibits the PLK1 signaling cascade and important cell cycle regulators at both mRNA and protein levels to block NB cell cycle progression (Figure 5) and to overall inhibit NB growth (Figure 1, Figure 2, Figure 6 and Figure 7).

## 3. Discussion

PLK1 is a mitotic kinase that regulates the cell cycle transmission from G2 to mitosis and progression through mitosis [25,26,27,28]. The expression and activity of PLK1 are reported to be low throughout the G0, G1, and S phases, whereas peak levels of PLK1 are reported during the mitotic phase [26,29,30]. Several PLK1 inhibitors, including TKM-080301 (NCT01437007), BI-2536 (NCT00710710), GSK461364 (NCT00536835), volasertib (NCT02905994), TAK-960 (NCT01179399), BI 6727 (NCT01348347, NCT01145885), CYC140 (NCT03884829), Rigosertib (NCT03786237), and NMS-1286937 (NCT01014429), are currently under clinical trials for the treatment of various cancers, including pancreatic, prostate, breast, non-small-cell lung, and head and neck cancers [31].The FDA has granted a fast-track designation for developing onvansertib to treat patients with KRAS-mutated metastatic colorectal cancer, in combination with bevacizumab and FOLFIRI (5-fluorouracil, leucovorin, and irinotecan) [32]. Some of the PLK1 inhibitors were withdrawn from clinical trials due to high toxicity-related issues [33]. HMN-214, a novel oral stilbene derivative of HMN-176, has been reported as a well-tolerated drug with minimal gastrointestinal and neuropathic toxicities during the phase I pharmacokinetic study [21].

Overexpression of PLK1 was also reported in the highly aggressive NB tumors [34]. We also found, by using the NB primary patient transcriptomic data, that high levels of PLK1 lead to the poor overall survival of NB patients and correlate with NB progression. Inhibition of PLK1 by PLK1 inhibitors or depletion of PLK1 by siRNA-mediated knockdown induced apoptosis and mitotic catastrophe in various cancers, including NB [10,35,36]. Similar to the previous studies, we also confirmed a dose-dependent induction of apoptosis and blockage of the cell cycle at the G2/M phase in NB cells in response to HMN-214 treatment. Previous research reports show that cancerous cells are more vulnerable to PLK1 inhibitors than healthy or normal cells [37]. We also observed a dose-dependent inhibition of NB cell proliferation, whereas the normal fibroblast cell lines showed the limited effect of HMN-214, suggesting that HMN-214 selectively inhibits NB cancer cell growth.

PLK1 mRNA expression is reported to be high in actively dividing cells and various cancer cells, including NB [10]. Additionally, PLK1 inhibitors were reported to reduce the mRNA expression of various cell cycle regulatory genes such as *CDK1*, *CDK2*, *CHK1, CHK2*, *Cyclin B1*, and *WEE1* [38,39,40]. Our results show a dose-dependent reduction in *PLK1* mRNA expression in NB cells in response to HMN-214 treatment. We also observed HMN-214-mediated inhibition of several cell cycle genes *CCNB1*, *CDK1*, *WEE1*, *CDK2*, *CHEK1*, and *CHEK2* in NB cells. PLK1 and MDM2 are the negative regulators of p53, and PLK1 inhibitors have been reported to reduce the interaction of MDM2 and p53, thereby stabilizing the levels of p53, leading to cellular senescence [37]. Inhibition of PLK1 by BI-2536 reduced the mRNA expression of MDM2 and restored the function of wild-type p53 in the adrenocortical carcinoma [37]. Similarly, we observed a dose-dependent reduction in *MDM2* mRNA levels in response to HMN-214 treatment in NB cells. Furthermore, we observed that HMN-214 directly inhibits the phosphorylation and activation of PLK1 and also inhibits cell cycle regulator CDK1 to block the NB cell cycle progression at the G2/M phase. We demonstrate that HMN-214 significantly inhibits NB growth in both 2D and 3D tumor models, probably by directly inhibiting PLK1 and other molecular targets.

## 4. Materials and Methods

### 4.1. Cell Culture, Reagents and Patient Dataset

Six human NB cell lines—including both MYCN-amplified (NGP, LAN-5, IMR-32) and MYCN-non-amplified (SH-SY5Y, SK-N-AS, CHLA-255) cell lines and three normal fibroblast control cell lines (WI-38, NIH-3T3, COS-7)—were routinely cultured and maintained as described previously [41]. All the cell lines used in this study were validated via short-tandem repeat analysis for genotyping within the last six months and were tested for mycoplasma monthly. Primary antibodies anti-PLK1 (627701), anti-pPLK1 (Thr210) (699956), and anti-CDK1 (Cdc2) (626901) were purchased from BioLegend, Inc. Anti-Cyclophilin B (43603S), and anti-rabbit IgG HRP-linked secondary antibodies (7074S) were purchased from Cell Signaling Technology. HMN-214 (HY-12045) was purchased from MedChem Express, NJ and diluted in DMSO (Dimethyl sulfoxide). Control groups shown in different figures in this study were treated with DMSO and used as vehicle control. Data from 1235 NB primary patients were analyzed (Versteeg dataset (*n* = 88), Kocak dataset (*n* = 649), and SEQC dataset (*n* = 498)), using the publicly available R2: Genomic Analysis and Visualization Platform. This dataset includes microarray profiles of unique primary tumors and supports multi-parametric analysis of NB patient outcomes with gene expression data.

### 4.2. Cell Viability and Colonogenic Assay

Cell viability assays were performed using MTT cell proliferation dye (3-(4,5-Dimethylthiazol-2-yl)-2,5-Diphenyltetrazolium Bromide) (L11939; Alfa Aesar). Briefly, 1 × 10^4^ cells per well were seeded into a 96-well plate and treated with different concentrations of HMN-214 for 72 h and developed as per the manufacturer’s instructions. Clonogenic assays were performed as described previously [42]. Colonies were stained and visualized using 0.2% crystal violet solution and counted using colony counting software. All assays were performed in triplicates and repeated at least thrice with proper controls. GraphPad Prism 9 software was used to analyze the data and calculate the IC_50_ values.

### 4.3. Apoptosis and Cell Cycle Assay

Apoptosis assays were performed using Muse Annexin V & Dead Cell Kit (MCH100105; Luminex Corp, Austin, DX, USA), and cell cycle assays were performed using Click-iT™ Plus EdU Alexa Fluor™ 488 Flow Cytometry Assay Kit (C10633; ThermoFisher Scientific, Waltham, MA, USA) and FxCycle™ PI/RNase Staining Solution (F10797; ThermoFisher Scientific) according to the manufacturer’s instructions and as described previously [41]. Briefly, 1 × 10^6^ NB cells per well were seeded in a six-well plate followed by treatment with different doses of HMN-214 for 16 h. Apoptosis assays were performed using Muse flow cytometer (Luminex) and cell cycle assays were performed using Attune Nxt Flow Cytometer (ThermoFisher Scientific). Data were analyzed using Muse and Flow Jo software version 10 (BD Biosciences).

### 4.4. 3D Spheroid Tumor Assay

Three-dimensional spheroidal 96-well microplates (4515; Corning, NY, USA) were used to perform 3D spheroidal tumor assays as described previously [41]. Briefly, 300 µm sized spheroids were randomly selected and treated with different concentrations of HMN-214. Spheroid drug treatment continued for 12 days with regular drug replenishment and spheroid tumor size measurement every third day. Spheroid tumor images were captured using a Leica DMi1 microscope, and the size was measured using the LASX software suite (Leica Microsystems, Wetzlar, Germany). On the 12th day, spheroids were fluorescent stained using Viability/Cytotoxicity Assay Kit for Animal Live & Dead Cells (3002; Biotium Inc., Fremont, CA, USA) as described earlier [42]. The fluorescence image was captured using the EVOS FL imaging system (Thermo Scientific, Waltham, MA, USA).

### 4.5. RNA Extraction and Quantitative Real-Time RT-PCR

Gene expression analysis was performed using the RT-qPCR analysis as described previously [41]. Briefly, 1 × 10^6^ NB cells per well were seeded into a 6-well plate and treated with the different concentrations of HMN-214 for 12 h. Total RNA was extracted using RNeasy plus mini kit (74134; Qiagen) according to manufactures instructions and as described previously [41]. High-capacity cDNA reverse transcription kit (4368814; ThermoFisher Scientific) was used to synthesize cDNA from total RNA according to manufactures instructions. Further, RT-qPCR reactions for individual genes were performed using SYBR Green dye (4385610; ThermoFisher Scientific) using the QuantStudio 3 Real-Time PCR System (ThermoFisher Scientific) as described previously [41]. Primers were designed using the NCBI primer design software and listed in Table 1. GAPDH was used as a housekeeping gene to normalize the expression of individual genes. All reactions were performed in triplicates and repeated three times. The *p* values were calculated by Student’s *t*-test for expression fold difference of individual genes. Primers used in this study are as follows.

### 4.6. Western Blot Analysis

Western blots were performed as described previously [42]. Briefly, 3 × 10^6^ NB cells were seeded in a 10 mm petri dish and treated with increasing concentrations of HMN-214 for 12 h. Cell pellets were collected and lysed with RIPA extraction buffer cocktail and the extracted protein was quantified using Bradford assay (5000205; Bio-Rad). Protein samples were loaded and separated on 12% SDS-PAGE gel and transferred to the PVDF membrane, blocked with 5% BSA solution and then probed with corresponding primary and secondary antibodies. Clarity ECL Western substrate (Bio-Rad) and ChemiDoc XRS Plus system (Bio-Rad) was used to develop, visualize, and document the blots. Protein bands were quantified using the ImageJ software. CyclophilinB (CyPB) was used as a loading and normalizing control.

## 5. Statistical Analysis

All the assays performed in the present study were repeated at least three times with three technical replicates. All values are presented as the mean ± standard deviation (SD). A two-tailed Student’s *t*-test was used to determine the statistical significance among drug treatment groups. *p* < 0.05 was considered statistically significant. Patient survival analyses were performed using the Kaplan–Meier method and two-sided log-rank tests.

## 6. Conclusions

In conclusion, our study demonstrates the importance of PLK1 in regulating NB progression and growth and highlights PLK1 as a target for developing effective NB therapeutic approaches. We also demonstrate that the repurposing of HMN-214 to inhibit PLK1 in NB is a novel therapeutic approach. HMN-214 significantly inhibits NB proliferation, induces apoptosis, inhibits cell cycle regulators, inhibits NB 3D spheroid tumor growth, and directly inhibits the phosphorylation and activation of PLK1 in NB to block the cell cycle progression at the G2/M phase. In our future efforts, we will determine the effects of other PLK1 inhibitors to further confirm the findings of the present study and to establish the role of PLK1 in NB. Overall, our preclinical data suggest repurposing of HMN-214 and combining it with current therapies to develop effective therapeutic approaches for children battling NB.

## Figures and Tables

**Table 1:** Primers.

Gene	Forward Primer (5′-3′)	Reverse Primer (5′-3′)
*PLK1*	AAGAGATCCCGGAGGTCCTA	TCATTCAGGAAAAGGTTGCC
*WEE1*	TGGAGATCAATGGCATGAAA	AGTGCCATTGCTGAAGGTCT
*CDK1*	CATGGATTCTTCACTTGTTAAGGT	TCCACTTCTGGCCACACTTC
*CDK2*	TGGACACGCTGCTGGATG	AATGGCAGAAAGCTAGGCCC
*c-Myc*	TACACTAACATCCCACGCTCTG	CGCATCCTTGTCCTGTGAGT
*CCBN1*	AAGAGCTTTAAACTTTGGTCTGGG	CTTTGTAAGTCCTTGATTTACCATG
*CHEK1*	GACTGGGACTTGGTGCAAAC	TGCCATGAGTTGATGGAAGA
*CHEK2*	TGAGAACCTTATGTGGAACCCC	ACAGCACGGTTATACCCAGC
*MDM2*	GCAGTGAATCTACAGGGACGC	ATCCTGATCCAACCAATCACC
*GAPDH*	CACCATCTTCCAGGAGCGAG	TGATGACCCTTTTGGCTCCC

## Data Availability

Data is contained within the article.
